# Multiple species delimitation approaches with *COI* barcodes poorly fit each other and morphospecies – An integrative taxonomy case of Sri Lankan Sericini chafers (Coleoptera: Scarabaeidae)

**DOI:** 10.1002/ece3.8942

**Published:** 2022-05-19

**Authors:** Uda Gedara Sasanka Lakmali Ranasinghe, Jonas Eberle, Jana Thormann, Claudia Bohacz, Suresh P. Benjamin, Dirk Ahrens

**Affiliations:** ^1^ Zoological Research Museum A. Koenig Leibniz Institute for the Analysis of Biodiversity Change (LIB) Bonn Germany; ^2^ University of Salzburg Salzburg Austria; ^3^ National Institute of Fundamental Studies Kandy Sri Lanka

**Keywords:** barcoding, integrative taxonomy, taxonomic match ratio

## Abstract

DNA taxonomy including barcoding and metabarcoding is widely used to explore the diversity in biodiversity hotspots. In most of these hotspot areas, chafers are represented by a multitude of species, which are well defined by the complex shape of male genitalia. Here, we explore how well *COI* barcode data reflect morphological species entities and thus their usability for accelerated species inventorization. We conducted dedicated field surveys in Sri Lanka to collect the species‐rich and highly endemic Sericini chafers (Coleoptera: Scarabaeidae). Congruence among results of a series of protocols for de novo species delimitation and with morphology‐based species identifications was investigated. Different delimitation methods, such as the Poisson tree processes (PTP) model, Statistical Parsimony Analysis (TCS), Automatic Barcode Gap Discovery (ABGD), Assemble Species by Automatic Partitioning (ASAP), and Barcode Index Number (BIN) assignments, resulted in different numbers of molecular operational taxonomic units (MOTUs). All methods showed both over‐splitting and lumping of morphologically identified species. Only 18 of the observed 45 morphospecies perfectly matched MOTUs from all methods. The congruence of delimitation between MOTUs and morphospecies expressed by the match ratio was low, ranging from 0.57 to 0.67. TCS and multirate PTP (mPTP) showed the highest match ratio, while (BIN) assignment resulted in the lowest match ratio and most splitting events. mPTP lumped more species than any other method. Principal coordinate analysis (PCoA) on a match ratio‐based distance matrix revealed incongruent outcomes of multiple DNA delimitation methods, although applied to the same data. Our results confirm that *COI* barcode data alone are unlikely to correctly delimit all species, in particular, when using only a single delimitation approach. We encourage the integration of various approaches and data, particularly morphology, to validate species boundaries.

## Graphical abstract

How well *COI* barcode data reflects species entities of morphologically well‐identified Sericini chafers as the study group in a diverse tropical hotspot. The study inferred the maximum likelihood trees represent the first molecular phylogenetic hypotheses for Sri Lankan Sericini.

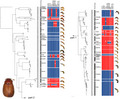

## 1 INTRODUCTION

Many regions on Earth that are exceptionally rich in endemic species are facing massive habitat loss (Costello et al., 2013). Most of those areas have been identified as “biodiversity hotspots” (Myers et al., 2000). In order to be able to conserve the vast diversity of currently largely unknown species, one necessity is to recognize them (Costello et al., 2013; Modica et al., 2014; Smith et al., 2005). For this purpose, DNA barcoding approaches have been widely used to explore diversity of both flora and fauna, especially in biodiversity hotspots where efficient conservation priorities are imperative (Barber & Boyce, 2006; Barman et al., 2018; Bezeng et al., 2017; Boissin et al., 2017; Geiger et al., 2014; Grosjean et al., 2015; Hebert et al., 2004; Hebert, Cywinska, et al., 2003; Hebert, Ratnasingham, et al., 2003; Jamaluddin et al., 2019; Kadarusman et al., 2012; Lahaye et al., 2008; Nagy et al., 2012; Oberprieler et al., 2018; Smith et al., 2005). These techniques attempt species delimitation and specimen identification based on a single‐gene fragment, e.g., from the *COI* gene. The state of the art, advantages and drawbacks, as well as their current usage have been extensively discussed in a number of works (Dellicour & Flot, 2018; DeSalle & Goldstein, 2019; Leliaert et al., 2014; Luo et al., 2018; Rannala & Yang, 2020; Vogler & Monaghan, 2007). Subsequently, approaches have been employed (metabarcoding), which allow large‐scale assessments of biodiversity through environmental DNA (Heyde et al., 2020; Hobern, 2021; Taberlet et al., 2012) in both terrestrial (Fernandes et al., 2018; Holdaway et al., 2017) and aquatic habitats (Leduc et al., 2019). With such metabarcoding approaches, it is not only possible to rapidly assess biodiversity but also to investigate external impacts on poorly studied invertebrate communities in highly diverse ecosystems (Dopheide et al., 2020; Vogler et al., 2021).

However, DNA barcoding also has been critically discussed since its first emergence due to many problems coming particularly from the nature of the used single mtDNA marker gene (Ballard & Whitlock, 2004; Eberle et al., 2020; Krishnamurthy & Francis, 2012). Many empirical studies investigated the robustness of DNA barcoding and the used species delimitation methods, particularly in the context of inherent natural bias of species such as fluctuating effective population size or unbalanced representation of specimen samples (Ahrens et al., 2016; Esselstyn et al., 2012; Fujisawa & Barraclough, 2013).

While results of *COI* barcoding have been so far mainly compared with morphospecies entities, the congruence of the outcome of different DNA‐based species delimitations has only rarely been analyzed in detail. Outcomes have often been characterized as “different” without quantifying the difference (Ahrens et al., 2016; Dalstein et al., 2019; Lukic et al., 2021). These differences are explored here in detail, exemplified by a case study of Sri Lankan Sericini chafers.

Sri Lanka, together with Southern Indian Western Ghats, is one of the world's outstanding biodiversity hotspots, harboring unique and threatened biota (Myers et al., 2000). So far, only a handful of larger barcoding studies have been conducted on the Indian subcontinent. These include the identification of disease vectors (Tabanid flies: Banerjee et al., 2015; sand flies: Gajapathy et al., 2016; biting midges Culicoides: Harrup et al., 2016; mosquitos: Weeraratne et al., 2018), and also of highly invasive agricultural pests (fruit fly: Khamis et al., 2012; tea mosquito bugs: Rebijith et al., 2012; Pentatomomorpha bugs: Tembe et al., 2014; Kaur & Sharma, 2016; thrips: Tyagi et al., 2017; and fall armyworm: Nanayakkara et al., 2020). Furthermore, barcoding approaches have been used to resolve taxonomic questions in butterflies (Goonesekera et al., 2019; Rajpoot et al., 2018), fishes (Dhaneesh et al., 2015; Ekanayake et al., 2021; Lakra et al., 2016; Raja & Perumal, 2017; Senevirathna & Munasinghe, 2013), frogs (Biju et al., 2014; Meegaskumbura et al., 2015), freshwater crabs (Beenaerts et al., 2010), spiders (Ileperuma Arachchi & Benjamin, 2019; Kanesharatnam & Benjamin, 2019), snakes (Pyron et al., 2013), and snails (Raheem et al., 2017). Concerted and comprehensive initiatives, which coordinate the sampling and data basing efforts, as known from Europe and northern America, for example, are yet missing.

For the highly diverse beetles, apart from a few isolated studies that were very limited in taxon sampling (Asha & Sinu, 2020; Dangalle et al., 2014), DNA taxonomy approaches including barcoding have not yet been applied in the Western Ghats hotspot. This is even true for herbivore scarab chafers, of which some species appear to be serious crop pests despite being highly endemic (Ahrens, 2004; Ahrens & Fabrizi, 2016). In the last decade, dozens of new herbivore scarab species have been discovered from the subcontinent, and Asia in general (Ahrens & Fabrizi, 2016; Ahrens et al., 2014a, 2014b, 2014c; Fabrizi & Ahrens, 2014; Liu et al., 2014a, 2014b, 2014c, 2015, 2016).

Given the great use of *COI* barcode data for biodiversity assessments (Arribas et al., 2020, 2021), we were interested in expanding the existing punctual assessments of DNA barcoding (Ahrens et al., 2016; Dalstein et al., 2019; Lukic et al., 2021) and in exploring how well *COI* barcode data reflect species entities in a understudied tropical hotspot. We chose Sericini chafer beetles (Coleoptera: Scarabaeidae: Melolonthinae) as example study group because species can be well defined by strongly differentiated male genitalia (Dalstein et al., 2019; Eberle et al., 2016). We performed dedicated field surveys in Sri Lanka and investigated the match of morphospecies with the entities inferred by commonly used species delimitation algorithms based on the sequenced *COI* barcode data. Such focused tests continue to be necessary to further develop our understanding of frequently employed taxonomic markers in different organism groups, particularly in the light of potential drawbacks for accuracy of newly emerging approaches such as metabarcoding or “exclusively *COI* barcode‐based species definitions” (Sharkey, Brown, et al., 2021; Sharkey, Janzen, et al., 2021).

## 2 MATERIALS AND METHODS

### 2.1 Specimen sampling

**FIGURE 1:**
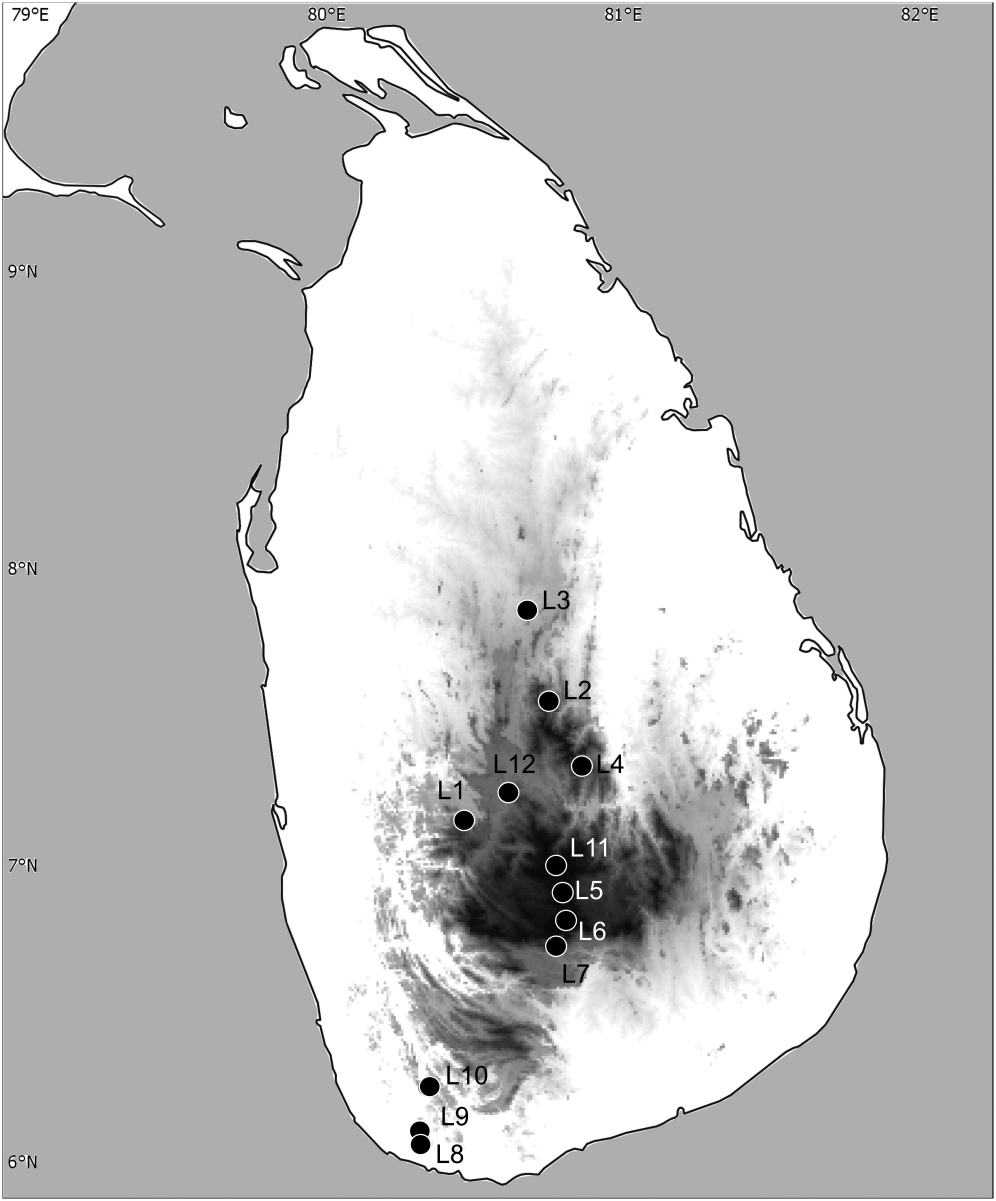
Map of Sri Lanka showing collecting sites for this study. IDs refer to major sampling localities: L1: Aranayake; L2: Riverston; L3: NIFS Arboretum; L4: Deenston; L5: Nuwara Eliya; L6: Horton Plains; 7: Belihuloya; L8: Hiyare; L9: Kottawa; L10: Kanneliya; L11: Piduruthalagala; L12: Uda Peradeniya

Sampling of adult Sericini chafers (Coleoptera: Scarabaeidae) was carried out at 12 localities in Sri Lanka (Figure 1) during 2019–2020. These sites included different forest types in different ecozones. Beetles were captured using ultraviolet‐light traps and manual collecting from a white sheet being illuminated with ultraviolet, blue, and green LEDs (LepiLED, © WIF, Dr Gunnar Brehm, Jena, Germany). Some additional specimens were hand collected during the day. All specimens were preserved in 96% ethanol after collecting.

The collected specimens were presorted to morphospecies. For this purpose, all male genitalia were dissected and labeled. Identification to species level was done using recent literature (Ahrens & Fabrizi, 2016; Fabrizi & Ahrens, 2014; Ranasinghe et al., 2020) and, in some cases, by comparison with type specimens. Three to seven male individuals of each morphospecies per location were selected for DNA extraction and subsequent sequencing (in total 280 individuals). The species’ habitus and male genitalia were photographed of one selected specimen per species, using a Zeiss AxioCam HRc camera (SteREO Discovery. V20). Images at several focal points were taken using the Zeiss Axio Vision (ZEN pro) software package and stacked with Zerene Stacker (Version 1.04) (http://www.zerenesystems.com).

### 2.2 DNA sequencing

DNA was extracted from mesothoracic leg and attached muscles using the Qiagen^®^ DNeasy Blood and Tissue Kit (Hilden, Germany) or the Qiagen^®^ BioSprint 96 magnetic bead extractor (Hilden, Germany). Lab work followed the standard protocols of the German Barcode of Life project (Geiger et al., 2016). The primers LCO1490‐JJ [5′‐CHACWAAYCATAAAGATATYGG‐3′] and HCO2198‐JJ [5′‐AWACTTCVGGRTGVCC AAARAATCA‐3′] (Astrin & Stüben, 2008) were used to amplify a 658 bp fragment at the 5'‐end of the mitochondrial gene cytochrome c oxidase subunit 1. PCRs of 90 samples were performed using the QIAGEN^®^ Multiplex PCR kit. The amplification products were subsequently checked by electrophoresis on a 1.5% agarose gel containing GelRed^®^. Successfully amplified DNA fragments were purified using Illustra™ ExoProStar™ Enzymatic PCR and Sequencing Clean‐Up kit. Forward and reverse strands were sequenced by Macrogen Europe (Macrogen; www.macrogen.com). PCRs for 190 samples were done in 96‐well plates. Unpurified PCR products were subsequently sent for purification and bidirectional Sanger sequencing to BGI Tech Solutions (Hongkong, China). Sequences were assembled, edited, and aligned using Geneious R7 (version 7.1.9, Biomatters Ltd.). All data are deposited in BOLD (project: SCOIB) and GenBank (accession numbers MW698204–MW698469) (see Table S1).

### 2.3 Phylogenetic analysis

Maximum likelihood (ML; Felsenstein, 1973) searches were performed in IQ‐TREE version 1.6.12 (Nguyen et al., 2015) under the (GTR+F+I+G4) model of nucleotide substitution that was inferred as the best‐fit model by ModelFinder (Kalyaanamoorthy et al., 2017). A total of 1000 ultrafast bootstrap (Hoang et al., 2018) replicates were done to assess branch supports. The tree search was repeated 10 times with the above parameters and the tree with highest likelihood was selected for further analysis. The resulting tree was rooted with *Apogonia* sp. (X‐SR0095) in FigTree v.1.4.4 (available from http://tree.bio.ed.ac.uk/software/figtree). Split networks as implemented in SplitsTree4 v.4.16.2 (available from http://www.splitstree.org) (Huson & Bryant, 2006) were used to represent incompatible and ambiguous signals in the *COI* dataset. Additionally, maximum likelihood searches were performed in PhyML using automatic model selection by Smart Model Selection (SMS) (Lefort et al., 2017) on the web server version (http://www.atgc‐montpellier.fr/phyml/) (Guindon et al., 2010).

### 2.4 Species delimitation

DNA‐based species delimitation was performed using the Poisson tree processes (PTP) model (Zhang et al., 2013), Statistical parsimony analysis (TCS) (Templeton, 2001; Templeton et al., 1992), Automatic Barcode Gap Discovery (ABGD) (Puillandre et al., 2012), Assemble Species by Automatic Partitioning (ASAP) (Puillandre et al., 2020), and Barcode Index Number (BIN) assignments (Ratnasingham & Hebert, 2013).

Poisson tree process modeling was performed with PTP web server (https://species.h‐its.org/; accessed on February 9, 2021) using the maximum likelihood implementation (hereafter mlPTP; Zhang et al., 2013) with a single Poisson distribution, as well as the Bayesian implementation (bPTP), which adds Bayesian support (pp) values for putative species to branches in the input tree. The PTP method infers speciation events based on a shift in the number of substitutions between internal nodes (Zhang et al., 2013).

Furthermore, multirate PTP (https://mptp.h‐its.org/#/tree; accessed on July 23, 2021) was performed. Multirate PTP (hereafter mPTP; Kapli et al., 2017) is an improved method of PTP which does not require user‐defined parameter as input and using MCMC it computes the support values for each clade, which can be used to assess the confidence of the ML delimitation. The IQ‐TREE result from previous phylogenetic analysis was used as input for all PTP analyses.

Statistical parsimony analysis was performed as implemented in TCS v.1.21 (Clement et al., 2000). The procedure partitions the sequence data into clusters, i.e., subgroups (or networks) of closely related haplotypes connected by changes with a <95% probability to be non‐homoplastic. Resulting networks have been found to be largely congruent with morphospecies at the 95% threshold (Ahrens et al., 2007; Meier et al., 2006) and are considered here as molecular operational taxonomic units (MOTUs).

Automatic Barcode Gap Discovery (ABGD) was conducted using the ABGD webserver (https://bioinfo.mnhn.fr/abi/public/abgd/abgdweb.html; accessed on February 9, 2021) with default parameters (i.e., using Jukes‐Cantor model (JC69) distances, a relative gap width of 1 and 50 steps, Pmin = 0.001, Pmax = 0.1, and Nb bins for distance distribution = 20) (Puillandre et al., 2012). ABGD applies a set of prior intraspecific divergences to detect the position of the barcode gap, which are iteratively refined. Alternatively, we reran the ABGD analysis with a distance matrix generated through IQ‐TREE analysis as the input file. This maximum likelihood distance values (mldist file) reflected pairwise distances corrected by the GTR model.

Assemble Species by Automatic Partitioning (ASAP) was conducted using the ASAP webserver (https://bioinfo.mnhn.fr/abi/public/asap/ accessed on July 23, 2021) using the distance matrix generated through IQ‐TREE analysis (Puillandre et al., 2020). ASAP divides species partitions based on pairwise genetic distances. ASAP also computes a probability of panmixia (*p*‐val), a relative gap width metric (W), and ranked results by the ASAP score: the lower the score, the better the partitioning (Puillandre et al., 2020). Number of MOTUs predicted by ASAP 1st and ASAP 2nd scores were selected and compared with other methods. Finally, MOTUs from Barcode Index Number (BIN) assignments (Ratnasingham & Hebert, 2013) obtained from the BOLD database (Project—SCOIB: Sericini *COI* Barcoding) were included and compared with other delimitation results.

**FIGURE 2:**
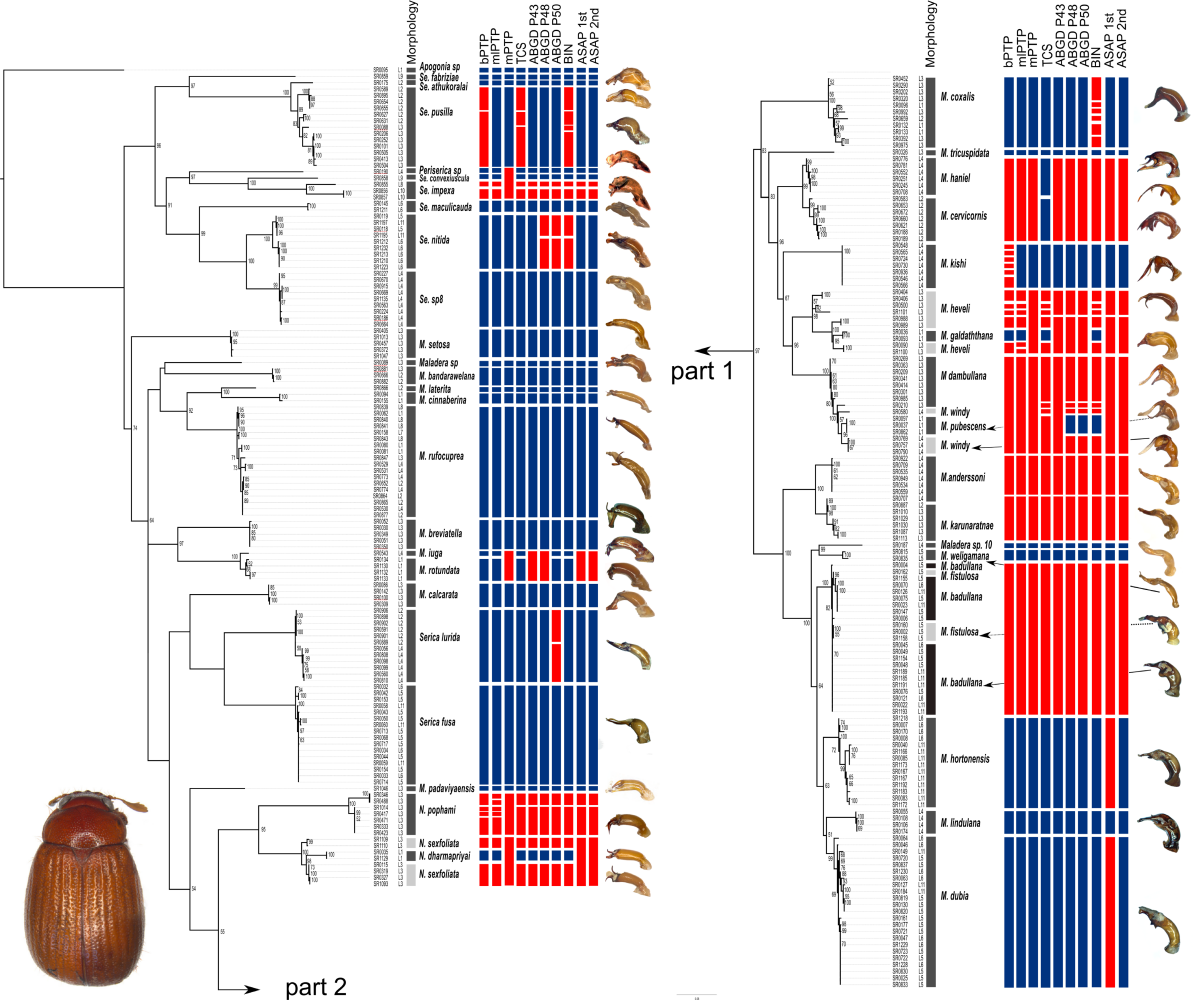
Maximum likelihood tree with information about morphospecies assignments, sampling locations, results of species delimitations (bPTP, mlPTP, mPTP, TCS, ABGD, BINs and ASAP), and illustrations of the respective morphospecies’ aedeagi in lateral view. Blue boxes indicate agreement between molecular species delimitation method and morphospecies assignment, while red boxes indicate disagreement. Ultrafast bootstrap supports (%) >50 are shown next to the branches

The accuracy of DNA‐based methods with prior morphospecies assignment was assessed by the match ratio (Ahrens et al., 2016): 2 × *N*_match_/(*N*_mol_ + *N*_morph_), where *N*_match_ is the number of exact matches of morphospecies (all individuals) with MOTUs of different delimitation methods, *N*_mol_ is the number of MOTUs that resulted from different delimitation methods, and *N*_morph_ is the number of morphospecies (Table 1). All morphospecies were mapped onto the terminals of the maximum likelihood tree along with the images of their male genitalia (lateral view) and MOTUs obtained from different species delimitation methods (Figure 2). Furthermore, the match ratios for all pairs of delimitation methods were calculated and compared in a similarity matrix. The matrix was transformed into a distance matrix and a principal coordinate analysis (PCoA) was performed in PAST v.3.25 (Hammer et al., 2001) in order to compare similarity between different methods.

**TABLE 1:** Match ratio (Ahrens et al., 2016) of DNA‐based species delimitation methods on Sericini chafer data based on number of MOTUs and number of matches between MOTUs and morphospecies (Nmorph = 45)

	bPTP	mlPTP	mPTP	TCS	ABGD P43	ABGD P48	ABGD P50	BIN	ASAP 1st	ASAP 2nd
N match	30	32	27	33	29	29	30	30	27	28
N MOTU	57	52	35	53	43	48	50	61	40	41
Match ratio	0.59	0.66	0.67	0.67	0.66	0.62	0.63	0.57	0.63	0.65

Note

Match ratio = 2 × *N*_match_/(*N*_mol_ + *N*_morph_).

### 2.5 Data handling

All raw data produced in the project are freely accessible and deposited in dedicated databases for secure and curated long‐term storage. Extracted DNA is stored at −80°C in the DNA bank collection of the ZFMK (https://www.zfmk.de/en/biobank). Obtained nucleotide sequences were submitted to NCBI (accession numbers MW698204–MW698469) and BOLD databases (project: SCOIB) (see Table S1). Voucher specimens are deposited in the insect collections of the ZFMK, each with a unique voucher ID that is linked to the corresponding DNA extract from that sample to the DNA sequences, and to the morphological data and images. Voucher‐related data and IDs were stored in the ZFMK’s digital collection database.

## 3 RESULTS

Morphological sorting of captured scarab specimens (total ca. 2300) resulted in a total of 45 Sericini morphospecies of which 280 individuals were selected for sequencing. These species included 41 Sri Lankan endemics and representatives from all five Sericini genera occurring in Sri Lanka. Thirty‐four morphospecies were represented by more than one individual and 11 were singletons. For 266 specimens, we successfully obtained *COI* sequences (658 bp). From 266 individuals, 257 were assigned to putative morphospecies using male genital preparations. Nine specimens were females which did not have suitable diagnostic characters for morphospecies assignment; they were subsequently assigned to species using the DNA sequences as they were clearly nested in the relevant species clades (seven specimens: SR0088, SR0100, SR0118, SR0186, SR0190, SR0350, and SR0881) or unambiguously assigned to a species in all delimitation methods (two specimens: SR0089 and SR0095). Despite our repeated extensive sampling (Figure 1), 75.5% of the taxa (34 morphospecies) were collected from only single localities. For example, nine species (20% of all recorded species) were only found at Deanston (L4), 10 species (22.2%) only at Dambulla (L3), and 6 species (13.3%) only at Aranayake (L1). However, 11 morphospecies (24.5% of all recorded species) were represented in more than one locality, but none was found in more than half of all localities: *Maladera badullana* (3 sites), *M*. *coxalis* (2 sites), *M*. *dubia* (3 sites), *M*. *hortonensis* (2 sites), *M*. *karunaratnae* (2 sites), *M*. *rufocuprea* (5 sites), *Serica fusa* (3 sites), *S*. *lurida* (2 sites), *Selaserica impexa* (2 sites), *Se*. *nitida* (2 sites), and *Se*. *pusilla* (2 sites).

ML tree searches with IQ‐TREE and PhyML obtained a similar tree topology (Figure 2, Figure [Link], [Link]), with the exception of three cases. With IQ‐TREE, *M*. *hortonensis* was sister to *M*. *dubia* +*M. lindulana*, whereas in the PhyML tree *M*. *lindulana* was sister to *M*. *dubia* + *M*. *hortonensis*. In the second case, *Maladera* sp. (female specimen, SR0089) was sister to *M*. *bandarawelana* in the IQ‐TREE tree and to *M*. *rotundata* + *M*. *igua* + *M*. *breviatella* in the PhyML tree. Finally, *M*. *igua* was nested within *M*. *rotundata* in the latter, whereas the IQ‐TREE resolved them as two sister species. However, several distinct clades were equally recovered in both trees, such as the clade *Selaserica* +Periserica, the *Serica* clade, the *Neoserica* clade, and the *Maladera fistulosa* clade. The latter is a diverse, endemic radiation on the island that is characterized by entirely reduced parameres. It included eight so far new, unnamed species, which will be described in a separate publication.

### 3.1 Species delimitation

The different species delimitation methods (bPTP, mlPTP, mPTP, TCS, ABGD, ASAP, and BIN) resulted in different numbers of MOTUs (Table 1). We found relatively limited congruence between molecular operational taxonomic units (MOTUs) and morphospecies. None of the employed species delimitation methods correctly inferred the same species partition that was obtained from prior morphospecies assignments. The number of MOTUs varied from 35 to 61.

Parsimony network analysis subdivided the unique haplotypes into 53 different MOTUs (i.e., networks). Thirty‐three of them perfectly matched with the morphospecies assignments and showed the highest, although moderate, match ratio of all delimitation methods (0.67). Most over‐splitting events could be attributed to larger geographical sequence variation.

Three different types of tree‐based PTP analyses (bPTP, mlPTP, and mPTP) resulted in varying numbers of MOTUs and matches with morphospecies. bPTP modeling showed low congruence, mainly due to over‐splitting, and resulted in 57 MOTUs with 30 matches with the morphospecies, displaying the second lowest match ratio (0.59). mlPTP modeling resulted in 52 MOTUs with 32 matches with the morphospecies and the second highest match ratio of 0.66. mPTP produced 35 MOTUs with 27 matches and the highest match ratio (0.67) similar to TCS analysis.

ABGD failed to identify a clear barcoding gap and thus resulted in unreliable results that strongly depended on parameter choice. No consistent estimate of the number of species was recovered across a range of initial parameter values. We arbitrarily chose three partitions with a prior maximal distance (P) of 0.018, 0.015, and 0.010 that resulted in 43, 48, and 50 MOTUs, respectively (hereafter as P43, P48, and P50), and matched with 29, 29, and 30 morphospecies. All three choices showed both lumping and splitting, and obtained match ratios between 0.63 and 0.66. The performance of ABGD thus lied between bPTP and TCS. The two best scores of ASAP partitioned species into groups containing 40 and 41 entities, and matched with 27 and 28 morphospecies, respectively. The resulting match ratio was 0.63 for the best scored partition, and 0.65 for the second partitioning. BIN assignments revealed 61 MOTUs and matched with 30 morphospecies. It obtained the lowest match ratio (0.57). BIN assignments showed more splitting events than any other method, for example, 6 MOTUs for 11 *M*. *coxalis* individuals collected from 2 different geographic locations, whereas all other methods resulted in a single MOTU for *M*. *coxalis*.

Only 18 MOTUs were obtained from all methods and also perfectly matched morphospecies. This included haplotypes from different geographic locations (e.g., in *Maladera rufocuprea* and *Serica fusa*). Thirty‐four morphospecies assignments entirely matched with MOTUs of at least one delimitation method. All methods showed both splitting (i.e., individuals of one morphospecies were separated into two or more different MOTUs) and lumping of morphospecies (i.e., individuals of two or more different morphospecies were joined in one MOTU) and produced different numbers of MOTUs and hence lower matching ratios (Table 1). Individuals of *Selaserica impexa* were split into different MOTUs according to their different geographic sampling locations with all delimitation methods except mPTP, while other species were only split by ABGD and BINs (*Se*. *nitida*), bPTP, BINs, and TCS (*Se*. *pusilla*) or ABGD (*S*. *lurida*).

A few non‐monophyletic species were observed: (1) *Neoserica dharmapriyai* was nested within *N*. *sexfoliata*; (2) *Maladera galdaththana* nested within *M*. *heveli*; (3) *M*. *pubescens* nested within *M*. *windy*; (4) one individual of *M*. *anderssoni* was placed in the *M*. *karunaratnae* clade; and (5) *M*. *badullana* and *M*. *fistulosa* were mixed within one clade. Consequently, individuals of *N*. *sexfoliata*, *M*. *heveli* and *M*. *windy* split into two or more MOTUs, while nested species were resolved as one MOTU or lumped with its sister species, resulting in low matches with morphospecies. In the first case, *N*. *sexfoliata* split into two MOTUs and *N*. *dharmapriyai* recovered as one separate MOTU that was nested within *N*. *sexfoliata* in all methods except mPTP and ASAP where both species were lumped into one MOTU. In the second case, *M*. *galdaththana* was lumped with the four individuals of *M*. *heveli* in ABGD and ASAP, however, mlPTP, bPTP, TCS, and BINs correctly assigned the species as a single MOTU. In mPTP, several but not all individuals of *M*. *galdaththana* and *M*. *heveli*, respectively, resulted as separate MOTUs. In the third case, all individuals of *M*. *pubescens*, *M*. *dambullana*, and *M*. *windy* were lumped together (bPTP, mlPTP, mPTP, and ASAP), whereas in TCS, ABGD, and BIN, additionally one individual from *M*. *dambullana* and *M*. *windy*, respectively, was split off resulting in two additional MOTUs. *M*. *pubescens* matched with prior morphospecies assignment in ABGD P48, ABGD P50, and BIN assignments. Both, fourth and fifth cases showed mixing of two different morphospecies: one individual of *M*. *anderssoni* (SR0707) was placed in the *M*. *karunaratnae* clade in all methods; moreover, *M*. *badullana* and *M*. *fistulosa* were mixed in all methods, thus both events resulted in lumping of species.

*M*. *cervicornis* and *M*. *haniel*, which differ very distinctly in shape of their male copulation organ, were lumped with all methods except in TCS. Whereas *M*. *iuga* and *M*. *rotundata* lumped in mPTP, ABGD, and ASAP, *M*. *dubia* showed lumping with *M*. *hortonensis* only in ASAP 1st partition. Over‐splitting was observed in *N*. *pophami* (SR0346 and SR0488 in mlPTP), *M*. *heveli* (SR0090 and SR1100 in mlPTP), and *M*. *kishi* (all individuals in bPTP) despite having identical sequences and being sampled from the same locality. All those cases affect matches with prior morphospecies assignments, hence decrease the match ratio in different delimitation methods.

**FIGURE 3:**
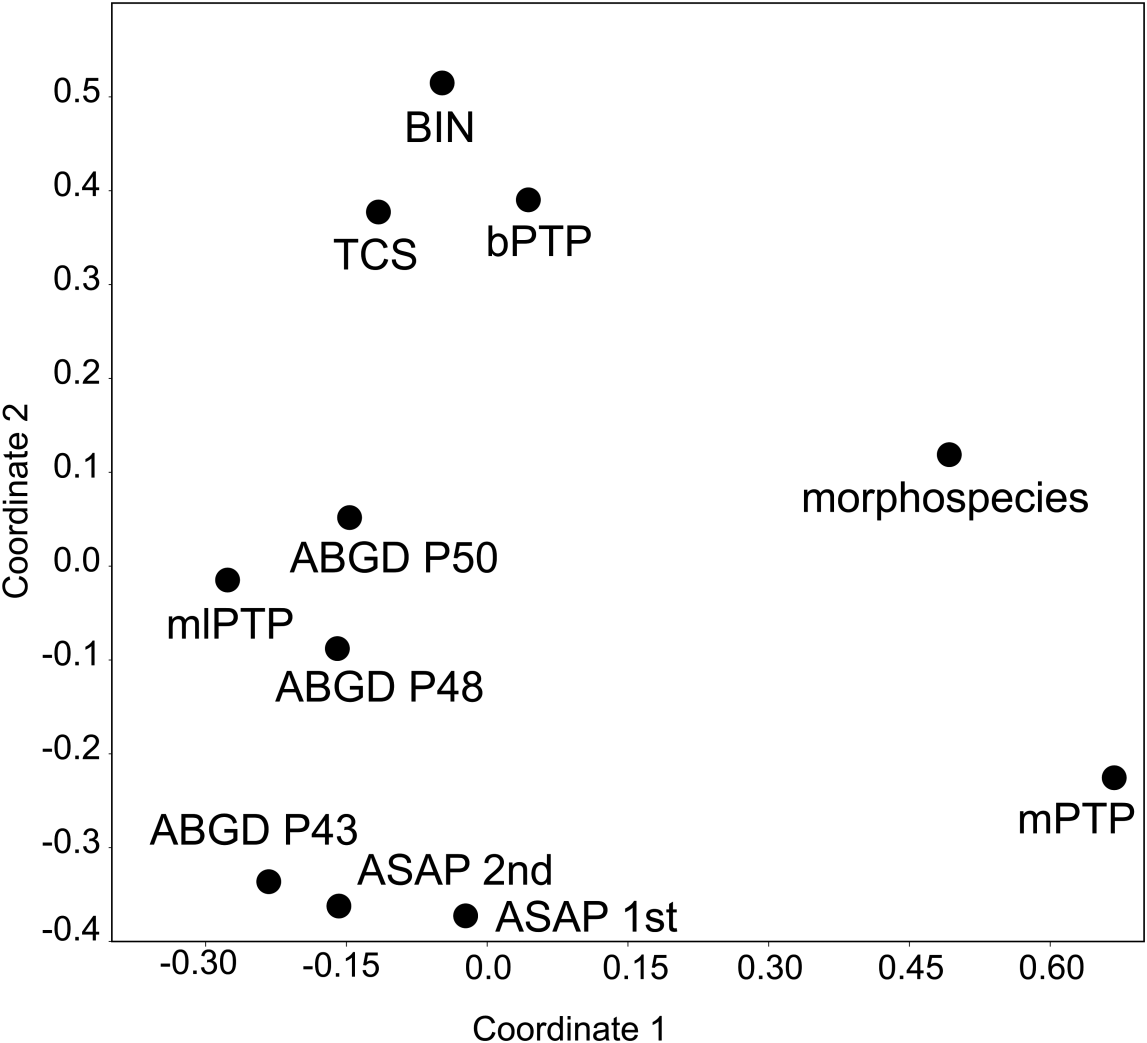
Principal coordinate analysis (PCoA) of different species delimitation methods and morphospecies based on pairwise match ratios

The PCoA ordination based on pairwise match ratios examined the similarity of the 10 different delimitation methods, which were all based on the same *COI* fragment, and also in relation to their congruence with morphology‐defined species (Figure 3). For *COI*‐based species delimitation, four distinct clusters were evident: one method resulted rather isolated (mPTP) and produced the lowest number of MOTUs (*n* = 35). TCS, BIN, and bPTP formed a second cluster; a third cluster consisted of ABGD P48, ABGD P50, and mlPTP, while ASAP 1st, ASAP 2nd and ABGD P43 formed the last one. These clusters correspond basically to the number of MOTUs of these methods (*N*_MOTU_ =53 –61, 48–52, and 40–43 for clusters 2–4, respectively) and they appear rather independent from prior morphospecies matches. This proposed ordination method can be used to show at one glance how different delimitation methods performed on a particular problem and to observe the similarity of *COI*‐based delimitation compared to that of morphology.

## 4 DISCUSSION

This study focused on the investigation of the performance of *COI* barcode data with various species delimitation approaches since *COI* barcodes are widely used as a proxy for species taxonomy and for ecological monitoring. Specifically, we investigated how well the resulting MOTUs reflected species entities in a megadiverse chafer group. While being rather uniform in external appearance, Sericini chafers show extremely well‐differentiated genitalia even between closely related species (Ahrens et al., 2016; Dalstein et al., 2019). The correlation between divergent genital morphology and evolutionary entities was widely confirmed by integrative taxonomy studies (Ahrens & Ribera, 2009; Eberle et al., 2016), including even genomic data (Dietz et al., 2021). The resulting maximum likelihood trees represent the first molecular phylogenetic hypotheses for Sri Lankan Sericini. Two distinct clades of *Selaserica* that were previously also characterized by morphological data (Fabrizi & Ahrens, 2014) could be confirmed in the present study. The two groups are characterized by the presence or absence of a carinate hypomeron for the *Selaserica splendifica* group and the *Se*. *nitida* group, respectively.

The here observed low congruence of MOTUs with morphospecies (match ratio: 0.57–0.67) was not unexpected since previous studies on tropical Sericini have showed similarly low match ratios (Ahrens et al., 2016; Dalstein et al., 2019; Lukic et al., 2021). Some of them were subject to strong variation (between 0.14 and 1) depending on the different selected subclades being analyzed (Ahrens et al., 2016). Even in the absence of geographic sampling bias, these have shown low match ratios (0.59–0.77) (Lukic et al., 2021). Also, the congruence between the different delimitation methods, although using the same data, was moderate. This is in line with graphical summaries of many DNA taxonomy studies that have shown rather inconsistent results among different species delimitation approaches using the same marker (see above; Bergsten et al., 2012; Magoga et al., 2021). However, some studies, particularly those with limited geographical (i.e., regional) scope in the northern hemisphere, showed almost perfect matches of MOTUs with morphospecies among nearly 90% of the studied species (Hendrich et al., 2015; Pentinsaari et al., 2014; Rulik et al., 2017). So far uninvestigated was their mutual multidimensional relations in terms of match ratios (Figure 3) (Ahrens et al., 2016). The observed divergent clusters in the plot of mutual match similarity, also in context with morphospecies, provided insight to the robustness and confidence of the results in a range of the used species delimitation approaches. Even using the same genetic marker, results differed conspicuously and call for caution regarding premature conclusions (Ahrens et al., 2021).

Inconsistency between *COI* delimited species and morphospecies can have different causes. In several cases, non‐monophyly of morphospecies was linked to splitting or lumping. Such cases were observed in the clades *M*. *fistulosa* group and in *Neoserica*. Non‐monophyly of species could be a result of introgression by hybridization or incomplete lineage sorting (ILS). Both phenomena can result in similar *COI* haplotypes across species boundaries and may consequently lead to splitting and/or lumping. If this occurs in morphologically highly dissimilar species (under the assumption that morphologically highly dissimilar species in terms of male genital shape are not closely related), it would rather indicate hybridization (Dalstein et al., 2019). In this study, hybridization may have occurred in case of *M*. *galdaththana* and *M*. *heveli*. Both taxa have highly dissimilar genitalia. In morphologically very similar species, non‐monophyly could be explained by either incomplete lineage sorting or introgressed DNA (Dalstein et al., 2019; Eberle et al., 2016). These cases were as follows: (1) *Neoserica dharmapriyai* and *N*. *sexfoliata*; (2) *M*. *pubescens*, *M*. *dambullana*, *and M*. *windy*; (3) *Maladera anderssoni* and *M*. *karunaratnae*; and (4) *M*. *badullana* and *M*. *fistulosa*. There are several tests to distinguish hybridization from incomplete lineage sorting (Joly et al., 2009; Sang & Zhong, 2000), which is, however, often difficult in reality (Eberle et al., 2016). Based on the available data (i.e., part of the *COI* gene), their application is impossible (Dalstein et al., 2019). Cross contamination of specimens during DNA extraction or PCR preparation could be excluded based on the position of the single samples in the microtiter plates, particularly for the case of *M*. *anderssoni* / *M*. *karunaratnae* (in which one individual of *M*. *anderssoni* was lumped with *M*. *karunaratnae* in all methods).

Inconsistency between MOTUs and morphospecies could have been caused by highly rapid speciation: *Maladera cervicornis* and *M*. *haniel*, which here both resulted as monophyletic sister taxa, have highly distinct male genitalia. They lumped in all methods except TCS. This could be indicative that divergence of their male genitalia is much faster and more distinct than mitochondrial molecular divergence and lineage sorting, which, although being complete, was not sufficient in terms of degree of divergence to delimit species unambiguously. Similar evidence for multiple species have been shown by Eberle et al. (2016) and confirmed with genomic data by Dietz et al. (2021).

Over‐splitting of morphospecies encountered here was apparently also caused by relatively deep coalescence, for example, by considerable geographically determined genetic variation in haplotypes (*Sel*. *impexa*). Specimens of this species originated from two isolated lowland forest reserves without any heterogeneous landscapes in between (L8 and L10; see Figure 1), so that individuals might not be able to migrate between these populations. Splitting of *Se*. *pusilla* (bPTP, TCS, BIN), *S*. *lurida* (ABGD), *M*. *coxalis* (BIN), and *Sel*. *nitida* (ABGD, BIN) also were determined by distant geographic sample locations of the specimens. *Maladera coxalis*, *Sel*. *nitida*, *Sel*. *pusilla*, and *S*. *lurida* were recorded from different forests in the central highlands with complex elevation patterns. A greater biodiversity is observed in these forests compared to lowland forests (Meegaskumbura et al., 2015), since they are separated by steep escarpments, gorges, parallel ridges, or peaks (Cooray, 1967), which may act as geographical barriers for dispersal and may result in partial reproductive isolation. However, spatial separation of individuals did not always cause over‐splitting. Morphospecies of *M*. *dubia*, *M*. *hortonensis*, *M*. *rufocuprea*, and *Serica fusa* were collected from different geographical locations and still appeared as a single entity in all analyses.

In general, as a result of limited dispersal, a negative relationship is expected for the geographic distance and the mating probability of individuals, as predicted by isolation by distance (Perez et al., 2018). Heterogeneous landscapes additionally might affect levels of gene flow due to reduced dispersal in consequence of the patchiness of preferred habitats (Perez et al., 2018; van Strien et al., 2015). Thus, it is obvious that low dispersal propensity contributes to an elevated but unknown extent of intraspecific, geographically structured divergence (Li et al., 2015). Alternatively, genetic divergence may also result from ecological adaptation or sexual selection (Boughman, 2001; Matsubayashi et al., 2013). What is actually more likely for each case under study is often unknown, as is the case of the Sri Lankan Sericini chafers studied here. Biased accumulation of mutations in mtDNA after population separation of widespread species can obscure the limits between putative species (Eberle et al., 2019). Restricted gene flow caused by large distances between populations can result in increased divergence (Bergsten et al., 2012; Perez et al., 2018) which can be exaggerated by (sex) biased or limited dispersal. In result, numbers of delimited entities can exceed the true species numbers by orders of magnitude (Eberle et al., 2019). Moreover, the effect of geography‐induced genetic divergence depends on the latitude as well as the involvement of diversity hotspots (Gaytán et al., 2020), which generally are also refugial areas (Ahrens et al., 2013), characterized by long‐term climatic stability (Fjeldså et al., 1999; Fjeldsaå et al., 1997; Harrison & Noss, 2017). All this would affect the output of different species delimitation methods and in fact, none of the used methods report accurate species numbers compared to prior morphospecies assignments.

Besides the above discussed issues like incomplete lineage sorting and introgression (Ballard & Whitlock, 2004; Funk & Omland, 2003), molecular species delimitation approaches based on information from a single gene such as the mitochondrial gene *COI* are frequently hampered by pseudogene co‐amplification or *Wolbachia* infections (Smith et al., 2012; Song et al., 2008), which may bias haplotype distributions. Furthermore, sampling size influences the results of delimitation methods (Ahrens et al., 2016; Luo et al., 2018). Low number of samples may affect species delimitation. Our sample size ranged from 5 to 10 individuals per species and sampling was the same for all delimitation methods, and therefore did not affect their comparison. The estimation of a tree topology also affects delimitation methods; relying on a single mitochondrial DNA marker system is prone to errors that can mislead species delimitation and identification (see Eberle et al., 2020).

The present study indicated that not the study organisms, i.e., the data itself, is the sole cause for incongruent species entities that were proposed by different methods. If signals were inherent to the data that caused mismatches with morphospecies, the same pattern of over‐splitting and lumping would be expected across all methods. This was not always the case (Figure 2). For example, lumping of *M*. *dubia* and *M*. *hortonensis* only in ASAP 1st partition, split of *M*. *coxlis* in BIN, and split of *S*. *lurida* in ABGD P50 (Figure 2). However, several species and species complexes were incongruent between morphospecies and several or all delimitation methods (e.g., *M*.* heveli and M*.* galdaththana* or *M*. *pubescens*, *M*. *dambullana*, *and M*. *windy*). We conclude that in these cases the used single marker system provided insufficient or misleading signal for accurate delimitation of species.

In order to bypass some of these difficulties of incongruence of morphospecies and species identification or delimitation with *COI* data, there have been proposals for a haplotype‐based macroecology, as patterns of intraspecific genetic diversity were found to be correlated with species richness (Papadopoulou et al., 2011) even at different spatial scales (Baselga et al., 2015). This way, highly valuable and easily produced data from, e.g., metabarcoding can be used (Taberlet et al., 2012). This becomes especially relevant in absence of complete reference barcode libraries in order to avoid high amounts of data deletion due to impossible species assignments.

Our results confirm that *COI* DNA barcode data alone are inadequate to delimit species, in particular in this case of Sericini chafers. Using various levels of haplotype diversity for ecological or evolutionary assessments bear high levels of uncertainty, as they might reflect different patterns at variable scales in time and space. However, these patterns appear to be not entirely and stringently evolutionary significant, as are those ones reflected by species. Thus, although haplotype diversity and species diversity are correlated, any ecological approach that does not take (true) species entities and species diversity in account might look at different ecological interrelationships and processes than those considering true species (Papadopoulou et al., 2011; Thormann et al., 2016). Also, our results strongly discourage approaches of a minimalist taxonomic procedure, defining (new) species based on *COI* barcode data alone, using a single species delimitation approach only without morphological reference diagnoses (Meierotto et al., 2019; Sharkey, Brown, et al., 2021; Sharkey, Janzen, et al., 2021) (see also Ahrens et al., 2021; Fernandez‐Triana, 2022; Meier et al., 2022; Zamani et al., 2021).

Due to the severe impact of human activities including climate change, numerous species risk going extinct before being discovered (Costello et al., 2013). An estimated 10 million species remain to be discovered (Dayrat, 2004). A stable and robust nomenclature is the basis of clear communication and scientific discussion about biodiversity. Including true species entities within biodiversity research incorporates evolutionary scales and processes at all time levels. In this manner, species entities and names provide the “anchor” to which all taxonomic, ecological, molecular, and conservation data are attached (International Trust for Zoological Nomenclature, 2008). Legal protection and policy are also linked to names (i.e., species), not to actual (mortal) individuals (or haplotypes), on the assumption that the groups indicated by the names are consistent through time and among places.

Conversely, recent integrative taxonomy studies revealed how difficult it actually is to infer species boundaries objectively and robustly and that, so far, no infallible method for species delimitation exists, even when using genomic data (Carstens et al., 2013; Rannala & Yang, 2020). New methods and data sources continue to being developed and examined empirically (Ahrens et al., 2016; Eberle et al., 2019; Fujisawa & Barraclough, 2013; Sukumaran & Knowles, 2017), gradually converging to detecting species boundaries (Dietz et al., 2021; Eberle et al., 2020; Rannala & Yang, 2020). However, issues of sampling and the inherent nature of species (e.g., the fluctuation of effective population size; Ahrens et al., 2016) are variables that will always impact large‐scale approaches and require continued integration with other sources of evidence (Padial et al., 2010; Schlick‐Steiner et al., 2010), thereby specifically accounting for characteristics of every single species (e.g., Campillo et al., 2020; Dufresnes et al., 2020; Hausdorf & Hennig, 2020).

## AUTHOR CONTRIBUTIONS

**Uda Gedara Sasanka Lakmali Ranasinghe:** Data curation (lead); Formal analysis (lead); Methodology (lead); Writing – original draft (lead); Writing – review & editing (supporting). **Jonas Eberle:** Conceptualization (supporting); Formal analysis (supporting); Methodology (supporting); Supervision (supporting); Writing – original draft (supporting); Writing – review & editing (supporting). **Jana Thormann:** Data curation (lead); Methodology (supporting). **Claudia Bohacz:** Methodology (supporting). **Suresh P. Benjamin:** Investigation (supporting); Resources (lead); Supervision (supporting). **Dirk Ahrens:** Conceptualization (lead); Data curation (lead); Formal analysis (lead); Funding acquisition (lead); Investigation (lead); Methodology (lead); Project administration (lead); Supervision (lead); Validation (lead); Writing – original draft (lead); Writing – review & editing (lead).

## CONFLICT OF INTEREST

We have no conflicts of interest to declare.

## Supporting information

Figure S1‐1

Figure S1‐2

Figure S2

Table S1

## Data Availability

DNA sequences: GenBank accessions (MW698204–MW698469); BOLD databases (project: SCOIB).
