# Supplementary figures and images for: Multiple species delimitation approaches with COI barcodes poorly fit each other and morphospecies – An integrative taxonomy case of Sri Lankan Sericini chafers (Coleoptera: Scarabaeidae)
Source: Ecol Evol. 2022 May 19;12(5):e8942. doi: 10.1002/ece3.8942 (PMC9120212; doi:10.1002/ece3.8942)

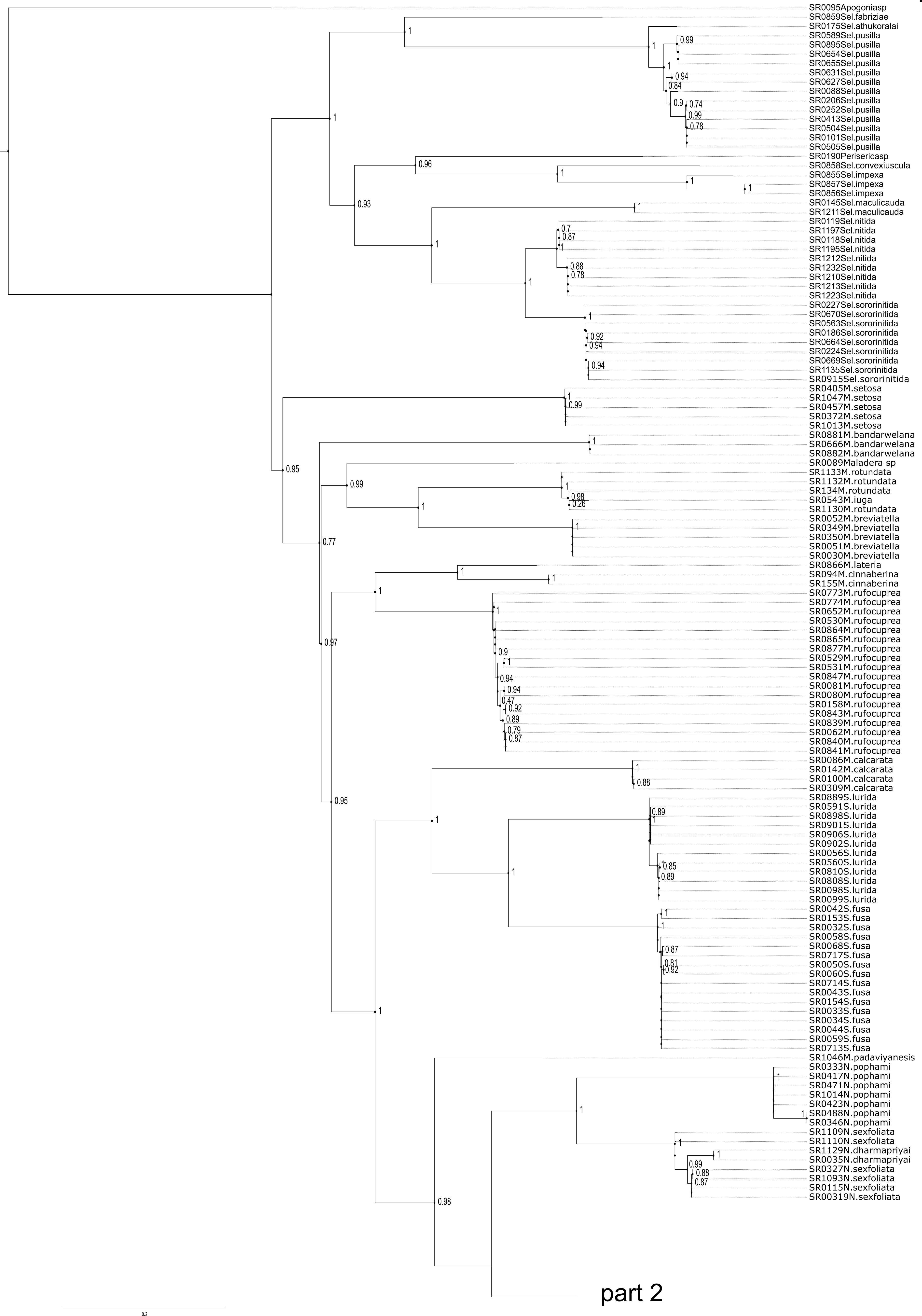

Supplement: Supplementary file 1 — Figure S1‐1 [file ECE3-12-e8942-s002.png]

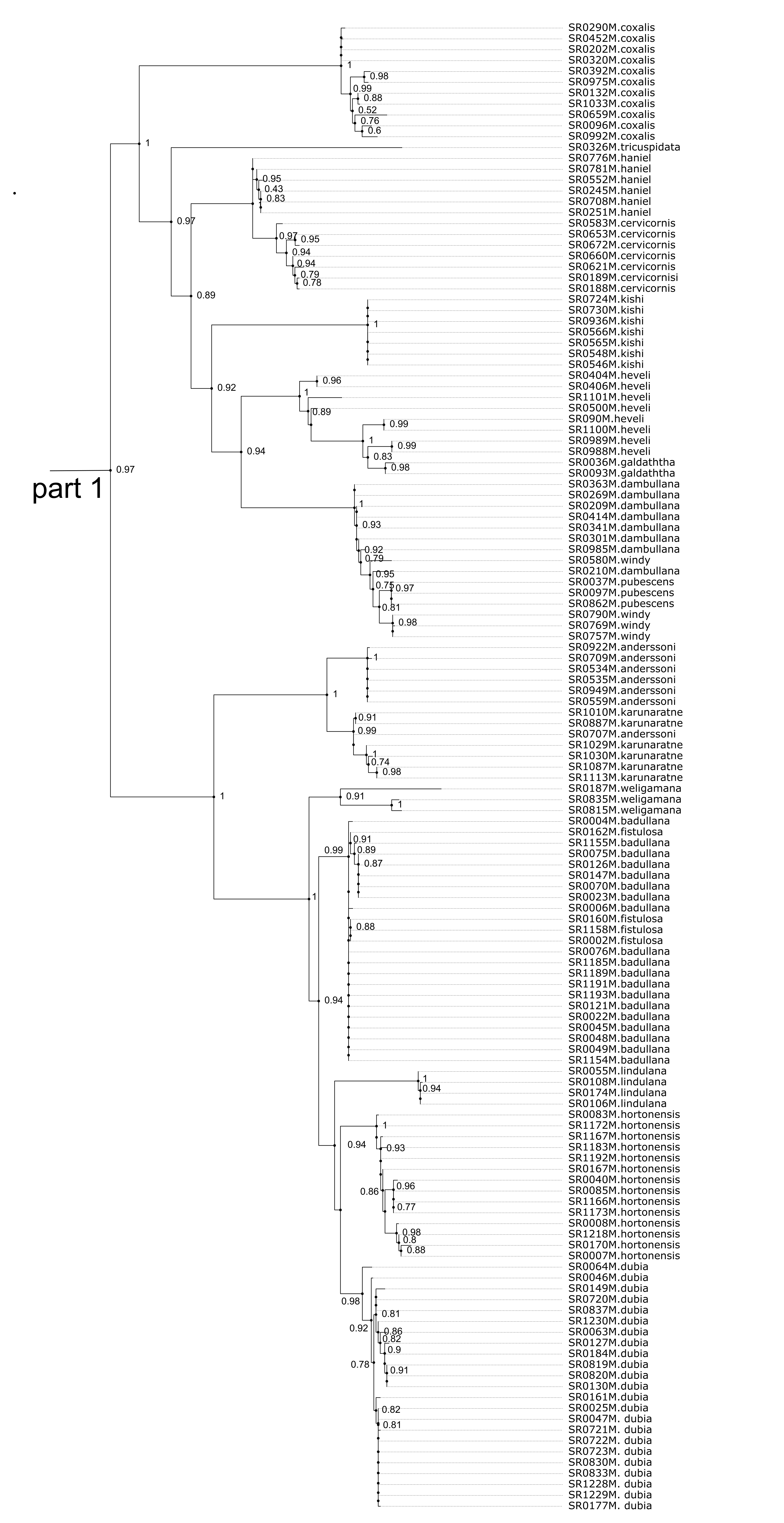

Supplement: Supplementary file 2 — Figure S1‐2 [file ECE3-12-e8942-s003.png]

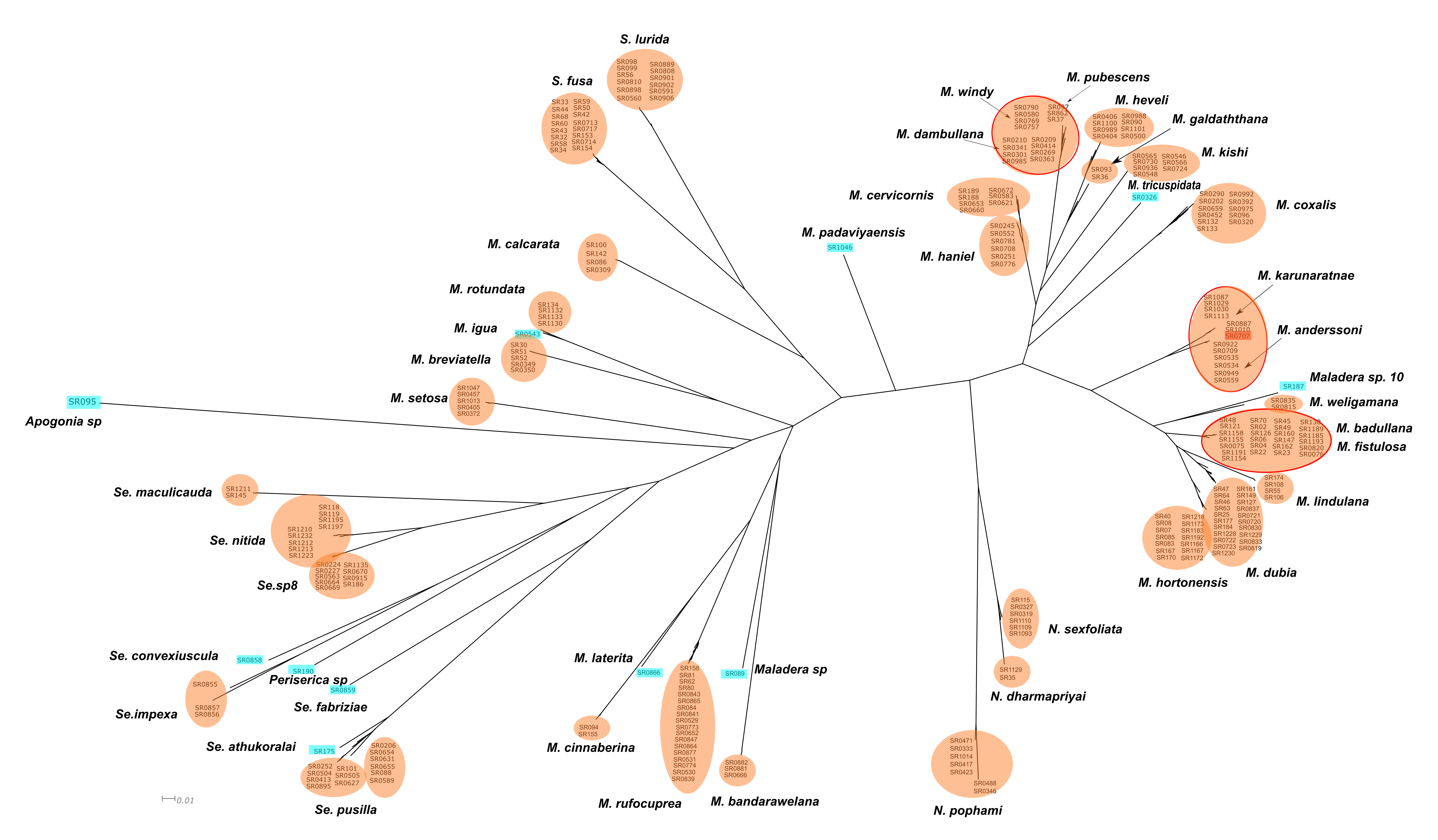

Supplement: Supplementary file 3 — Figure S2 [file ECE3-12-e8942-s004.png]
