# Supplementary material for: Multiple species delimitation approaches with COI barcodes poorly fit each other and morphospecies – An integrative taxonomy case of Sri Lankan Sericini chafers (Coleoptera: Scarabaeidae)
Source: Ecol Evol. 2022 May 19;12(5):e8942. doi: 10.1002/ece3.8942 (PMC9120212; doi:10.1002/ece3.8942)
Supplement: Supplementary file 4 — Table S1 [file ECE3-12-e8942-s001.docx]

Supplement Table 01

Table S1. Details of species; Voucher numbers, Species identification, sampling location (Sri Lanka) details with L numbers, Barcode Index Number (BIN) assignments and GenBank accession numbers.

| **Voucher ID** | **Species** | **District** | **Location** | **L Number** | **BIN** | **GenBank** |
| --- | --- | --- | --- | --- | --- | --- |
| **X-SR0002** | *Maladera fistulosa* | Nuwara Eliya District | Hakgala SNR | L5 | BOLD:AEH7081 | MW698428 |
| **X-SR0004** | *Maladera badullana* | Nuwara Eliya District | Hakgala SNR | L5 | BOLD:AEH7081 | MW698377 |
| **X-SR0006** | *Maladera badullana* | Nuwara Eliya District | Hakgala SNR | L5 | BOLD:AEH7081 | MW698281 |
| **X-SR0007** | *Maladera hortonensis* | Nuwara Eliya District | Horton Plains | L6 | BOLD:AEH5976 | MW698406 |
| **X-SR0008** | *Maladera hortonensis* | Nuwara Eliya District | Horton Plains | L6 | BOLD:AEH5976 | MW698437 |
| **X-SR0022** | *Maladera badullana* | Nuwara Eliya District | Piduruthalagala FR | L11 | BOLD:AEH7081 | MW698340 |
| **X-SR0023** | *Maladera badullana* | Nuwara Eliya District | Piduruthalagala FR | L11 | BOLD:AEH7081 | MW698379 |
| **X-SR0025** | *Maladera dubia* | Nuwara Eliya District | Hakgala SNR | L5 | BOLD:AEH6135 | MW698210 |
| **X-SR0030** | *Maladera breviatella* | Matale District | Dambulla | L3 | BOLD:AEH4423 | MW698320 |
| **X-SR0032** | *Serica fusa* | Nuwara Eliya District | Horton Plains | L6 | BOLD:AEH5844 | MW698451 |
| **X-SR0033** | *Serica fusa* | Nuwara Eliya District | Horton Plains | L6 | BOLD:AEH5844 | MW698455 |
| **X-SR0034** | *Serica fusa* | Nuwara Eliya District | Horton Plains | L6 | BOLD:AEH5844 | MW698351 |
| **X-SR0035** | *Neoserica dharmapriyai* | Kegalle District | Aranayake | L1 | BOLD:AEH7083 | MW698308 |
| **X-SR0036** | *Maladera galdaththana* | Kegalle District | Aranayake | L1 | BOLD:AEH4344 | MW698332 |
| **X-SR0037** | *Maladera pubescens* | Kegalle District | Aranayake | L1 | BOLD:AEH3996 | MW698417 |
| **X-SR0040** | *Maladera hortonensis* | Nuwara Eliya District | Piduruthalagala FR | L11 | BOLD:AEH5976 | MW698234 |
| **X-SR0042** | *Serica fusa* | Nuwara Eliya District | Hakgala SNR | L5 | BOLD:AEH5844 | MW698355 |
| **X-SR0043** | *Serica fusa* | Nuwara Eliya District | Hakgala SNR | L5 | BOLD:AEH5844 | MW698368 |
| **X-SR0044** | *Serica fusa* | Nuwara Eliya District | Hakgala SNR | L5 | BOLD:AEH5844 | MW698253 |
| **X-SR0045** | *Maladera badullana* | Nuwara Eliya District | Horton Plains | L6 | BOLD:AEH7081 | MW698260 |
| **X-SR0046** | *Maladera dubia* | Nuwara Eliya District | Horton Plains | L6 | BOLD:AEH6135 | MW698245 |
| **X-SR0047** | *Maladera dubia* | Nuwara Eliya District | Horton Plains | L6 | BOLD:AEH6135 | MW698391 |
| **X-SR0048** | *Maladera badullana* | Nuwara Eliya District | Galways Land NP | L5 | BOLD:AEH7081 | MW698432 |
| **X-SR0049** | *Maladera badullana* | Nuwara Eliya District | Galways Land NP | L5 | BOLD:AEH7081 | MW698330 |
| **X-SR0050** | *Serica fusa* | Nuwara Eliya District | Galways Land NP | L5 | BOLD:AEH5844 | MW698229 |
| **X-SR0051** | *Maladera breviatella* | Matale District | Dambulla | L3 | BOLD:AEH4423 | MW698362 |
| **X-SR0052** | *Maladera breviatella* | Matale District | Dambulla | L3 | BOLD:AEH4423 | MW698285 |
| **X-SR0055** | *Maladera lindulana* | Kandy District | Deenston | L4 | BOLD:AEH5722 | MW698359 |
| **X-SR0056** | *Serica lurida* | Kandy District | Deenston | L4 | BOLD:AEH5425 | MW698214 |
| **X-SR0058** | *Serica fusa* | Nuwara Eliya District | Piduruthalagala FR | L11 | BOLD:AEH5844 | MW698387 |
| **X-SR0059** | *Serica fusa* | Nuwara Eliya District | Piduruthalagala FR | L11 | BOLD:AEH5844 | MW698354 |
| **X-SR0060** | *Serica fusa* | Nuwara Eliya District | Piduruthalagala FR | L11 | BOLD:AEH5844 | MW698381 |
| **X-SR0062** | *Maladera rufocuprea* | Kegalle District | Aranayake | L1 | BOLD:AEH5150 | MW698220 |
| **X-SR0063** | *Maladera dubia* | Nuwara Eliya District | Horton Plains | L6 | BOLD:AEH6135 | MW698468 |
| **X-SR0064** | *Maladera dubia* | Nuwara Eliya District | Horton Plains | L6 | BOLD:AEH6135 | MW698257 |
| **X-SR0068** | *Serica fusa* | Nuwara Eliya District | Hakgala SNR | L5 | BOLD:AEH5844 | MW698321 |
| **X-SR0070** | *Maladera badullana* | Nuwara Eliya District | Horton Plains | L6 | BOLD:AEH7081 | MW698326 |
| **X-SR0080** | *Maladera rufocuprea* | Kegalle District | Aranayake | L1 | BOLD:AEH5150 | MW698336 |
| **X-SR0081** | *Maladera rufocuprea* | Kegalle District | Aranayake | L1 | BOLD:AEH5150 | MW698353 |
| **X-SR0083** | *Maladera hortonensis* | Nuwara Eliya District | Piduruthalagala FR | L11 | BOLD:AEH5976 | MW698408 |
| **X-SR0085** | *Maladera hortonensis* | Nuwara Eliya District | Piduruthalagala FR | L11 | BOLD:AEH5976 | MW698211 |
| **X-SR0086** | *Maladera calcarata* | Matale District | Dambulla | L3 | BOLD:AEH6472 | MW698448 |
| **X-SR0088** | *Selaserica sp* | Matale District | Dambulla | L3 | BOLD:AEH5331 | MW698227 |
| **X-SR0089** | *Maladera sp* | Matale District | Dambulla | L3 | BOLD:AEH7067 | MW698383 |
| **X-SR0090** | *Maladera heveli* | Matale District | Dambulla | L3 | BOLD:AEH8286 | MW698205 |
| **X-SR0093** | *Maladera galdaththana* | Kegalle District | Aranayake | L1 | BOLD:AEH4344 | MW698404 |
| **X-SR0094** | *Maladera cinnaberina* | Kegalle District | Aranayake | L1 | BOLD:AEH7181 | MW698389 |
| **X-SR0095** | *Apogonia sp* | Kegalle District | Aranayake | L1 | BOLD:AEH3418 | MW698424 |
| **X-SR0096** | *Maladera coxalis* | Kegalle District | Aranayake | L1 | BOLD:AEH6262 | MW698392 |
| **X-SR0097** | *Maladera pubescens* | Kegalle District | Aranayake | L1 | BOLD:AEH3996 | MW698233 |
| **X-SR0098** | *Serica lurida* | Kandy District | Deenston | L4 | BOLD:AEH5425 | MW698366 |
| **X-SR0099** | *Serica lurida* | Kandy District | Deenston | L4 | BOLD:AEH5425 | MW698446 |
| **X-SR0100** | *Maladera calcarata* | Matale District | Dambulla | L3 | BOLD:AEH6472 | MW698277 |
| **X-SR0101** | *Selaserica pusilla* | Matale District | Dambulla | L3 | BOLD:AEH5010 | MW698291 |
| **X-SR0106** | *Maladera lindulana* | Kandy District | Deenston | L4 | BOLD:AEH5722 | MW698415 |
| **X-SR0108** | *Maladera lindulana* | Kandy District | Deenston | L4 | BOLD:AEH5722 | MW698370 |
| **X-SR0115** | *Neoserica sexfoliata* | Matale District | Dambulla | L3 | BOLD:AEH7100 | MW698293 |
| **X-SR0118** | *Selaserica sp* | Nuwara Eliya District | Hakgala SNR | L5 | BOLD:AEH6994 | MW698467 |
| **X-SR0119** | *Selaserica nitida* | Nuwara Eliya District | Hakgala SNR | L5 | BOLD:AEH6994 | MW698331 |
| **X-SR0121** | *Maladera badullana* | Nuwara Eliya District | Horton Plains | L6 | BOLD:AEH7081 | MW698243 |
| **X-SR0126** | *Maladera badullana* | Nuwara Eliya District | Piduruthalagala FR | L11 | BOLD:AEH7081 | MW698375 |
| **X-SR0127** | *Maladera dubia* | Nuwara Eliya District | Piduruthalagala FR | L11 | BOLD:AEH6135 | MW698235 |
| **X-SR0130** | *Maladera dubia* | Nuwara Eliya District | Hakgala SNR | L5 | BOLD:AEH6135 | MW698419 |
| **X-SR0132** | *Maladera coxalis* | Kegalle District | Aranayake | L1 | BOLD:AEH6264 | MW698334 |
| **X-SR0133** | *Maladera coxalis* | Kegalle District | Aranayake | L1 | BOLD:AEH6264 | MW698426 |
| **X-SR0134** | *Maladera rotundata* | Kegalle District | Aranayake | L1 | BOLD:AEH5377 | MW698276 |
| **X-SR0142** | *Maladera calcarata* | Matale District | Dambulla | L3 | BOLD:AEH6472 | MW698454 |
| **X-SR0145** | *Selaserica maculicauda* | Nuwara Eliya District | Horton Plains | L6 | BOLD:AEH4591 | MW698462 |
| **X-SR0147** | *Maladera badullana* | Nuwara Eliya District | Galways Land NP | L5 | BOLD:AEH7081 | MW698342 |
| **X-SR0149** | *Maladera dubia* | Nuwara Eliya District | Piduruthalagala FR | L11 | BOLD:AEH6135 | MW698259 |
| **X-SR0153** | *Serica fusa* | Nuwara Eliya District | Hakgala SNR | L5 | BOLD:AEH5844 | MW698294 |
| **X-SR0154** | *Serica fusa* | Nuwara Eliya District | Hakgala SNR | L5 | BOLD:AEH5844 | MW698439 |
| **X-SR0155** | *Maladera cinnaberina* | Kegalle District | Aranayake | L1 | BOLD:AEH7181 | MW698317 |
| **X-SR0158** | *Maladera rufocuprea* | Ratnapura District | Belihuloya | L7 | BOLD:AEH5150 | MW698263 |
| **X-SR0160** | *Maladera fistulosa* | Nuwara Eliya District | Hakgala SNR | L5 | BOLD:AEH7081 | MW698398 |
| **X-SR0161** | *Maladera dubia* | Nuwara Eliya District | Hakgala SNR | L5 | BOLD:AEH6135 | MW698322 |
| **X-SR0162** | *Maladera fistulosa* | Nuwara Eliya District | Hakgala SNR | L5 | BOLD:AEH7081 | MW698266 |
| **X-SR0167** | *Maladera hortonensis* | Nuwara Eliya District | Piduruthalagala FR | L11 | BOLD:AEH5976 | MW698206 |
| **X-SR0170** | *Maladera hortonensis* | Nuwara Eliya District | Horton Plains | L6 | BOLD:AEH5976 | MW698367 |
| **X-SR0174** | *Maladera lindulana* | Kandy District | Deenston | L4 | BOLD:AEH5722 | MW698345 |
| **X-SR0175** | *Selaserica athukoralai* | Matale District | Riverston | L2 | BOLD:AEH8417 | MW698298 |
| **X-SR0177** | *Maladera dubia* | Nuwara Eliya District | Hakgala SNR | L5 | BOLD:AEH6135 | MW698420 |
| **X-SR0184** | *Maladera dubia* | Nuwara Eliya District | Piduruthalagala FR | L11 | BOLD:AEH6135 | MW698403 |
| **X-SR0186** | *Selaserica sp* | Kandy District | Deenston | L4 | BOLD:AEH7391 | MW698445 |
| **X-SR0187** | *Maladera* sp. 10 | Kandy District | Deenston | L4 | BOLD:AEH7198 | MW698230 |
| **X-SR0188** | *Maladera cervicornis* | Matale District | Riverston | L2 | BOLD:AEH8443 | MW698244 |
| **X-SR0189** | *Maladera cervicornis* | Matale District | Riverston | L2 | BOLD:AEH8443 | MW698453 |
| **X-SR0190** | *Periserica sp* | Kandy District | Deenston | L4 | BOLD:AEH6212 | MW698382 |
| **X-SR0202** | *Maladera coxalis* | Matale District | Dambulla | L3 | BOLD:AEH8752 | MW698443 |
| **X-SR0206** | *Selaserica pusilla* | Matale District | Dambulla | L3 | BOLD:AEH5010 | MW698231 |
| **X-SR0209** | *Maladera dambullana* | Matale District | Dambulla | L3 | BOLD:AEH7262 | MW698226 |
| **X-SR0210** | *Maladera dambullana* | Matale District | Dambulla | L3 | BOLD:AEH3995 | MW698315 |
| **X-SR0224** | *Selaserica* sp. 8 | Kandy District | Deenston | L4 | BOLD:AEH7391 | MW698223 |
| **X-SR0227** | *Selaserica* sp. 8 | Kandy District | Deenston | L4 | BOLD:AEH7391 | MW698349 |
| **X-SR0245** | *Maladera haniel* | Kandy District | Deenston | L4 | BOLD:AEH8443 | MW698440 |
| **X-SR0251** | *Maladera haniel* | Kandy District | Deenston | L4 | BOLD:AEH8443 | MW698254 |
| **X-SR0252** | *Selaserica pusilla* | Matale District | Dambulla | L3 | BOLD:AEH5010 | MW698221 |
| **X-SR0269** | *Maladera dambullana* | Matale District | Dambulla | L3 | BOLD:AEH7262 | MW698449 |
| **X-SR0290** | *Maladera coxalis* | Matale District | Dambulla | L3 | BOLD:AEH8752 | MW698288 |
| **X-SR0301** | *Maladera dambullana* | Matale District | Dambulla | L3 | BOLD:AEH7262 | MW698361 |
| **X-SR0309** | *Maladera calcarata* | Matale District | Dambulla | L3 | BOLD:AEH6472 | MW698438 |
| **X-SR0319** | *Neoserica sexfoliata* | Matale District | Dambulla | L3 | BOLD:AEH7100 | MW698452 |
| **X-SR0320** | *Maladera coxalis* | Matale District | Dambulla | L3 | BOLD:AEH8752 | MW698337 |
| **X-SR0326** | *Maladera tricuspidata* | Matale District | Dambulla | L3 | BOLD:AEH5496 | MW698225 |
| **X-SR0327** | *Neoserica sexfoliata* | Matale District | Dambulla | L3 | BOLD:AEH7100 | MW698251 |
| **X-SR0333** | *Neoserica pophami* | Matale District | Dambulla | L3 | BOLD:AEH4365 | MW698239 |
| **X-SR0341** | *Maladera dambullana* | Matale District | Dambulla | L3 | BOLD:AEH7262 | MW698447 |
| **X-SR0346** | *Neoserica pophami* | Matale District | Dambulla | L3 | BOLD:AEH4366 | MW698287 |
| **X-SR0349** | *Maladera breviatella* | Matale District | Dambulla | L3 | BOLD:AEH4423 | MW698397 |
| **X-SR0350** | *Maladera breviatella* | Matale District | Dambulla | L3 | BOLD:AEH4423 | MW698215 |
| **X-SR0363** | *Maladera dambullana* | Matale District | Dambulla | L3 | BOLD:AEH7262 | MW698289 |
| **X-SR0372** | *Maladera setosa* | Matale District | Dambulla | L3 | BOLD:AEH6169 | MW698402 |
| **X-SR0392** | *Maladera coxalis* | Matale District | Dambulla | L3 | BOLD:AEH6964 | MW698255 |
| **X-SR0404** | *Maladera heveli* | Matale District | Dambulla | L3 | BOLD:AEH4245 | MW698459 |
| **X-SR0405** | *Maladera setosa* | Matale District | Dambulla | L3 | BOLD:AEH6169 | MW698358 |
| **X-SR0406** | *Maladera heveli* | Matale District | Dambulla | L3 | BOLD:AEH4245 | MW698297 |
| **X-SR0413** | *Selaserica pusilla* | Matale District | Dambulla | L3 | BOLD:AEH5010 | MW698457 |
| **X-SR0414** | *Maladera dambullana* | Matale District | Dambulla | L3 | BOLD:AEH7262 | MW698363 |
| **X-SR0417** | *Neoserica pophami* | Matale District | Dambulla | L3 | BOLD:AEH4365 | MW698378 |
| **X-SR0423** | *Neoserica pophami* | Matale District | Dambulla | L3 | BOLD:AEH4365 | MW698280 |
| **X-SR0452** | *Maladera coxalis* | Matale District | Dambulla | L3 | BOLD:AEH8752 | MW698463 |
| **X-SR0457** | *Maladera setosa* | Matale District | Dambulla | L3 | BOLD:AEH6169 | MW698286 |
| **X-SR0471** | *Neoserica pophami* | Matale District | Dambulla | L3 | BOLD:AEH4365 | MW698212 |
| **X-SR0488** | *Neoserica pophami* | Matale District | Dambulla | L3 | BOLD:AEH4366 | MW698373 |
| **X-SR0500** | *Maladera heveli* | Matale District | Dambulla | L3 | BOLD:AEH4244 | MW698319 |
| **X-SR0504** | *Selaserica pusilla* | Matale District | Dambulla | L3 | BOLD:AEH5010 | MW698312 |
| **X-SR0505** | *Selaserica pusilla* | Matale District | Dambulla | L3 | BOLD:AEH5010 | MW698344 |
| **X-SR0529** | *Maladera rufocuprea* | Kandy District | Deenston | L4 | BOLD:AEH5150 | MW698301 |
| **X-SR0530** | *Maladera rufocuprea* | Kandy District | Deenston | L4 | BOLD:AEH5150 | MW698328 |
| **X-SR0531** | *Maladera rufocuprea* | Kandy District | Deenston | L4 | BOLD:AEH5150 | MW698461 |
| **X-SR0534** | *Maladera anderssoni* | Kandy District | Deenston | L4 | BOLD:AEH8444 | MW698299 |
| **X-SR0535** | *Maladera anderssoni* | Kandy District | Deenston | L4 | BOLD:AEH8444 | MW698284 |
| **X-SR0543** | *Maladera igua* | Kandy District | Deenston | L4 | BOLD:AEH8370 | MW698441 |
| **X-SR0546** | *Maladera kishi* | Kandy District | Deenston | L4 | BOLD:AEH4589 | MW698265 |
| **X-SR0548** | *Maladera kishi* | Kandy District | Deenston | L4 | BOLD:AEH4589 | MW698218 |
| **X-SR0552** | *Maladera haniel* | Kandy District | Deenston | L4 | BOLD:AEH8443 | MW698219 |
| **X-SR0559** | *Maladera anderssoni* | Kandy District | Deenston | L4 | BOLD:AEH8444 | MW698216 |
| **X-SR0560** | *Serica lurida* | Kandy District | Deenston | L4 | BOLD:AEH5425 | MW698249 |
| **X-SR0563** | *Selaserica* sp. 8 | Kandy District | Deenston | L4 | BOLD:AEH7391 | MW698335 |
| **X-SR0565** | *Maladera kishi* | Kandy District | Deenston | L4 | BOLD:AEH4589 | MW698264 |
| **X-SR0566** | *Maladera kishi* | Kandy District | Deenston | L4 | BOLD:AEH4589 | MW698460 |
| **X-SR0580** | *Maladera windy* | Kandy District | Deenston | L4 | BOLD:AEH5536 | MW698303 |
| **X-SR0583** | *Maladera cervicornis* | Matale District | Riverston | L2 | BOLD:AEH8443 | MW698271 |
| **X-SR0589** | *Selaserica pusilla* | Matale District | Riverston | L2 | BOLD:AEH5009 | MW698365 |
| **X-SR0591** | *Serica lurida* | Matale District | Riverston | L2 | BOLD:AEH5425 | MW698425 |
| **X-SR0621** | *Maladera cervicornis* | Matale District | Riverston | L2 | BOLD:AEH8443 | MW698341 |
| **X-SR0627** | *Selaserica pusilla* | Matale District | Riverston | L2 | BOLD:AEH5008 | MW698393 |
| **X-SR0631** | *Selaserica pusilla* | Matale District | Riverston | L2 | BOLD:AEH5008 | MW698302 |
| **X-SR0652** | *Maladera rufocuprea* | Matale District | Riverston | L2 | BOLD:AEH5150 | MW698390 |
| **X-SR0653** | *Maladera cervicornis* | Matale District | Riverston | L2 | BOLD:AEH8443 | MW698405 |
| **X-SR0654** | *Selaserica pusilla* | Matale District | Riverston | L2 | BOLD:AEH5009 | MW698343 |
| **X-SR0655** | *Selaserica pusilla* | Matale District | Riverston | L2 | BOLD:AEH5009 | MW698217 |
| **X-SR0659** | *Maladera coxalis* | Matale District | Riverston | L2 | BOLD:AEH6236 | MW698427 |
| **X-SR0660** | *Maladera cervicornis* | Matale District | Riverston | L2 | BOLD:AEH8443 | MW698246 |
| **X-SR0664** | *Selaserica* sp. 8 | Kandy District | Deenston | L4 | BOLD:AEH7391 | MW698433 |
| **X-SR0666** | *Maladera bandarawelana* | Matale District | Riverston | L2 | BOLD:AEH5956 | MW698309 |
| **X-SR0669** | *Selaserica* sp. 8 | Kandy District | Deenston | L4 | BOLD:AEH7391 | MW698292 |
| **X-SR0670** | *Selaserica* sp. 8 | Kandy District | Deenston | L4 | BOLD:AEH7391 | MW698395 |
| **X-SR0672** | *Maladera cervicornis* | Matale District | Riverston | L2 | BOLD:AEH8443 | MW698327 |
| **X-SR0707** | *Maladera anderssoni* | Kandy District | Deenston | L4 | BOLD:AEH8445 | MW698348 |
| **X-SR0708** | *Maladera haniel* | Kandy District | Deenston | L4 | BOLD:AEH8443 | MW698307 |
| **X-SR0709** | *Maladera anderssoni* | Kandy District | Deenston | L4 | BOLD:AEH8444 | MW698305 |
| **X-SR0713** | *Serica fusa* | Nuwara Eliya District | Hakgala SNR | L5 | BOLD:AEH5844 | MW698296 |
| **X-SR0714** | *Serica fusa* | Nuwara Eliya District | Hakgala SNR | L5 | BOLD:AEH5844 | MW698385 |
| **X-SR0717** | *Serica fusa* | Nuwara Eliya District | Hakgala SNR | L5 | BOLD:AEH5844 | MW698372 |
| **X-SR0720** | *Maladera dubia* | Nuwara Eliya District | Hakgala SNR | L5 | BOLD:AEH6135 | MW698431 |
| **X-SR0721** | *Maladera dubia* | Nuwara Eliya District | Hakgala SNR | L5 | BOLD:AEH6135 | MW698418 |
| **X-SR0722** | *Maladera dubia* | Nuwara Eliya District | Hakgala SNR | L5 | BOLD:AEH6135 | MW698273 |
| **X-SR0723** | *Maladera dubia* | Nuwara Eliya District | Hakgala SNR | L5 | BOLD:AEH6135 | MW698252 |
| **X-SR0724** | *Maladera kishi* | Kandy District | Deenston | L4 | BOLD:AEH4589 | MW698283 |
| **X-SR0730** | *Maladera kishi* | Kandy District | Deenston | L4 | BOLD:AEH4589 | MW698436 |
| **X-SR0757** | *Maladera windy* | Kandy District | Deenston | L4 | BOLD:AEH3994 | MW698369 |
| **X-SR0769** | *Maladera windy* | Kandy District | Deenston | L4 | BOLD:AEH3994 | MW698347 |
| **X-SR0773** | *Maladera rufocuprea* | Kandy District | Deenston | L4 | BOLD:AEH5150 | MW698400 |
| **X-SR0774** | *Maladera rufocuprea* | Kandy District | Deenston | L4 | BOLD:AEH5150 | MW698429 |
| **X-SR0776** | *Maladera haniel* | Kandy District | Deenston | L4 | BOLD:AEH8443 | MW698421 |
| **X-SR0781** | *Maladera haniel* | Kandy District | Deenston | L4 | BOLD:AEH8443 | MW698414 |
| **X-SR0790** | *Maladera windy* | Kandy District | Deenston | L4 | BOLD:AEH3994 | MW698456 |
| **X-SR0808** | *Serica lurida* | Kandy District | Deenston | L4 | BOLD:AEH5425 | MW698300 |
| **X-SR0810** | *Serica lurida* | Kandy District | Deenston | L4 | BOLD:AEH5425 | MW698236 |
| **X-SR0815** | *Maladera weligamana* | Nuwara Eliya District | Hakgala SNR | L5 | BOLD:AEH7197 | MW698339 |
| **X-SR0819** | *Maladera dubia* | Nuwara Eliya District | Hakgala SNR | L5 | BOLD:AEH6135 | MW698222 |
| **X-SR0820** | *Maladera dubia* | Nuwara Eliya District | Hakgala SNR | L5 | BOLD:AEH6135 | MW698356 |
| **X-SR0830** | *Maladera dubia* | Nuwara Eliya District | Hakgala SNR | L5 | BOLD:AEH6135 | MW698401 |
| **X-SR0833** | *Maladera dubia* | Nuwara Eliya District | Hakgala SNR | L5 | BOLD:AEH6135 | MW698469 |
| **X-SR0835** | *Maladera weligamana* | Nuwara Eliya District | Hakgala SNR | L5 | BOLD:AEH7197 | MW698310 |
| **X-SR0837** | *Maladera dubia* | Nuwara Eliya District | Hakgala SNR | L5 | BOLD:AEH6135 | MW698325 |
| **X-SR0839** | *Maladera rufocuprea* | Galle District | Hiyare FR | L8 | BOLD:AEH5150 | MW698306 |
| **X-SR0840** | *Maladera rufocuprea* | Galle District | Hiyare FR | L8 | BOLD:AEH5150 | MW698338 |
| **X-SR0841** | *Maladera rufocuprea* | Galle District | Hiyare FR | L8 | BOLD:AEH5150 | MW698399 |
| **X-SR0843** | *Maladera rufocuprea* | Galle District | Hiyare FR | L8 | BOLD:AEH5150 | MW698407 |
| **X-SR0847** | *Maladera rufocuprea* | Galle District | Hiyare FR | L8 | BOLD:AEH5150 | MW698275 |
| **X-SR0855** | *Selaserica impexa* | Galle District | Hiyare FR | L8 | BOLD:AEH7222 | MW698290 |
| **X-SR0856** | *Selaserica impexa* | Galle District | Kanneliya FR | L10 | BOLD:AEH7223 | MW698413 |
| **X-SR0857** | *Selaserica impexa* | Galle District | Kanneliya FR | L10 | BOLD:AEH7223 | MW698333 |
| **X-SR0858** | *Selaserica convexiuscula* | Galle District | Kottawa FR | L9 | BOLD:AEH6645 | MW698268 |
| **X-SR0859** | *Selaserica fabriziae* | Galle District | Kottawa FR | L9 | BOLD:AEH6644 | MW698316 |
| **X-SR0862** | *Maladera pubescens* | Kegalle District | Aranayake | L1 | BOLD:AEH3996 | MW698272 |
| **X-SR0864** | *Maladera rufocuprea* | Matale District | Riverston | L2 | BOLD:AEH5150 | MW698282 |
| **X-SR0865** | *Maladera rufocuprea* | Matale District | Riverston | L2 | BOLD:AEH5150 | MW698444 |
| **X-SR0866** | *Maladera laterita* | Matale District | Riverston | L2 | BOLD:AEH6029 | MW698465 |
| **X-SR0877** | *Maladera rufocuprea* | Matale District | Riverston | L2 | BOLD:AEH5150 | MW698207 |
| **X-SR0881** | *Maladera bandarawelana* | Matale District | Riverston | L2 | BOLD:AEH5956 | MW698374 |
| **X-SR0882** | *Maladera bandarawelana* | Matale District | Riverston | L2 | BOLD:AEH5956 | MW698466 |
| **X-SR0887** | *Maladera karunaratnae* | Matale District | Riverston | L2 | BOLD:AEH8445 | MW698237 |
| **X-SR0889** | *Serica lurida* | Matale District | Riverston | L2 | BOLD:AEH5425 | MW698422 |
| **X-SR0895** | *Selaserica pusilla* | Matale District | Riverston | L2 | BOLD:AEH5009 | MW698208 |
| **X-SR0898** | *Serica lurida* | Matale District | Riverston | L2 | BOLD:AEH5425 | MW698240 |
| **X-SR0901** | *Serica lurida* | Matale District | Riverston | L2 | BOLD:AEH5425 | MW698371 |
| **X-SR0902** | *Serica lurida* | Matale District | Riverston | L2 | BOLD:AEH5425 | MW698270 |
| **X-SR0906** | *Serica lurida* | Matale District | Riverston | L2 | BOLD:AEH5425 | MW698304 |
| **X-SR0915** | *Selaserica* sp. 8 | Kandy District | Deenston | L4 | BOLD:AEH7391 | MW698278 |
| **X-SR0922** | *Maladera anderssoni* | Kandy District | Deenston | L4 | BOLD:AEH8444 | MW698450 |
| **X-SR0936** | *Maladera kishi* | Kandy District | Deenston | L4 | BOLD:AEH4589 | MW698213 |
| **X-SR0949** | *Maladera anderssoni* | Kandy District | Deenston | L4 | BOLD:AEH8444 | MW698396 |
| **X-SR0975** | *Maladera coxalis* | Matale District | Dambulla | L3 | BOLD:AEH6964 | MW698311 |
| **X-SR0985** | *Maladera dambullana* | Matale District | Dambulla | L3 | BOLD:AEH7262 | MW698380 |
| **X-SR0988** | *Maladera heveli* | Matale District | Dambulla | L3 | BOLD:AEH5052 | MW698410 |
| **X-SR0989** | *Maladera heveli* | Matale District | Dambulla | L3 | BOLD:AEH5052 | MW698224 |
| **X-SR0992** | *Maladera coxalis* | Matale District | Dambulla | L3 | BOLD:AEH6263 | MW698241 |
| **X-SR1010** | *Maladera karunaratnae* | Matale District | Dambulla | L3 | BOLD:AEH8445 | MW698232 |
| **X-SR1013** | *Maladera setosa* | Matale District | Dambulla | L3 | BOLD:AEH6169 | MW698238 |
| **X-SR1014** | *Neoserica pophami* | Matale District | Dambulla | L3 | BOLD:AEH4365 | MW698256 |
| **X-SR1029** | *Maladera karunaratnae* | Matale District | Dambulla | L3 | BOLD:AEH8445 | MW698394 |
| **X-SR1030** | *Maladera karunaratnae* | Matale District | Dambulla | L3 | BOLD:AEH8445 | MW698247 |
| **X-SR1046** | *Maladera padaviyaensis* | Matale District | Dambulla | L3 | BOLD:AEH6028 | MW698209 |
| **X-SR1047** | *Maladera setosa* | Matale District | Dambulla | L3 | BOLD:AEH6169 | MW698423 |
| **X-SR1087** | *Maladera karunaratnae* | Matale District | Dambulla | L3 | BOLD:AEH8445 | MW698258 |
| **X-SR1093** | *Neoserica sexfoliata* | Matale District | Dambulla | L3 | BOLD:AEH7100 | MW698435 |
| **X-SR1100** | *Maladera heveli* | Matale District | Dambulla | L3 | BOLD:AEH8286 | MW698204 |
| **X-SR1101** | *Maladera heveli* | Matale District | Dambulla | L3 | BOLD:AEH8287 | MW698248 |
| **X-SR1109** | *Neoserica sexfoliata* | Matale District | Dambulla | L3 | BOLD:AEH7101 | MW698458 |
| **X-SR1110** | *Neoserica sexfoliata* | Matale District | Dambulla | L3 | BOLD:AEH7101 | MW698411 |
| **X-SR1113** | *Maladera karunaratnae* | Matale District | Dambulla | L3 | BOLD:AEH8445 | MW698442 |
| **X-SR1129** | *Neoserica dharmapriyai* | Kegalle District | Aranayake | L1 | BOLD:AEH7083 | MW698360 |
| **X-SR1130** | *Maladera rotundata* | Kegalle District | Aranayake | L1 | BOLD:AEH5377 | MW698262 |
| **X-SR1132** | *Maladera rotundata* | Kegalle District | Aranayake | L1 | BOLD:AEH5377 | MW698329 |
| **X-SR1133** | *Maladera rotundata* | Kegalle District | Aranayake | L1 | BOLD:AEH5377 | MW698388 |
| **X-SR1135** | *Selaserica* sp. 8 | Kandy District | Deenston | L4 | BOLD:AEH7391 | MW698324 |
| **X-SR1154** | *Maladera badullana* | Nuwara Eliya District | Hakgala SNR | L5 | BOLD:AEH7081 | MW698416 |
| **X-SR1155** | *Maladera badullana* | Nuwara Eliya District | Hakgala SNR | L5 | BOLD:AEH7081 | MW698228 |
| **X-SR1158** | *Maladera fistulosa* | Nuwara Eliya District | Hakgala SNR | L5 | BOLD:AEH7081 | MW698350 |
| **X-SR1166** | *Maladera hortonensis* | Nuwara Eliya District | Piduruthalagala FR | L11 | BOLD:AEH5976 | MW698267 |
| **X-SR1167** | *Maladera hortonensis* | Nuwara Eliya District | Piduruthalagala FR | L11 | BOLD:AEH5976 | MW698314 |
| **X-SR1172** | *Maladera hortonensis* | Nuwara Eliya District | Piduruthalagala FR | L11 | BOLD:AEH5976 | MW698430 |
| **X-SR1173** | *Maladera hortonensis* | Nuwara Eliya District | Piduruthalagala FR | L11 | BOLD:AEH5976 | MW698242 |
| **X-SR1183** | *Maladera hortonensis* | Nuwara Eliya District | Piduruthalagala FR | L11 | BOLD:AEH5976 | MW698376 |
| **X-SR1185** | *Maladera badullana* | Nuwara Eliya District | Piduruthalagala FR | L11 | BOLD:AEH7081 | MW698346 |
| **X-SR1189** | *Maladera badullana* | Nuwara Eliya District | Piduruthalagala FR | L11 | BOLD:AEH7081 | MW698357 |
| **X-SR1191** | *Maladera badullana* | Nuwara Eliya District | Piduruthalagala FR | L11 | BOLD:AEH7081 | MW698386 |
| **X-SR1192** | *Maladera hortonensis* | Nuwara Eliya District | Piduruthalagala FR | L11 | BOLD:AEH5976 | MW698318 |
| **X-SR1193** | *Maladera badullana* | Nuwara Eliya District | Piduruthalagala FR | L11 | BOLD:AEH7081 | MW698279 |
| **X-SR1195** | *Selaserica nitida* | Nuwara Eliya District | Piduruthalagala FR | L11 | BOLD:AEH6994 | MW698261 |
| **X-SR1197** | *Selaserica nitida* | Nuwara Eliya District | Piduruthalagala FR | L11 | BOLD:AEH6994 | MW698384 |
| **X-SR1210** | *Selaserica nitida* | Nuwara Eliya District | Horton Plains | L6 | BOLD:AEH6993 | MW698364 |
| **X-SR1211** | *Selaserica maculicauda* | Nuwara Eliya District | Horton Plains | L6 | BOLD:AEH4591 | MW698313 |
| **X-SR1212** | *Selaserica nitida* | Nuwara Eliya District | Horton Plains | L6 | BOLD:AEH6993 | MW698434 |
| **X-SR1213** | *Selaserica nitida* | Nuwara Eliya District | Horton Plains | L6 | BOLD:AEH6993 | MW698409 |
| **X-SR1218** | *Maladera hortonensis* | Nuwara Eliya District | Horton Plains | L6 | BOLD:AEH5976 | MW698352 |
| **X-SR1223** | *Selaserica nitida* | Nuwara Eliya District | Horton Plains | L6 | BOLD:AEH6993 | MW698412 |
| **X-SR1228** | *Maladera dubia* | Nuwara Eliya District | Horton Plains | L6 | BOLD:AEH6135 | MW698295 |
| **X-SR1229** | *Maladera dubia* | Nuwara Eliya District | Horton Plains | L6 | BOLD:AEH6135 | MW698323 |
| **X-SR1230** | *Maladera dubia* | Nuwara Eliya District | Horton Plains | L6 | BOLD:AEH6135 | MW698274 |
| **X-SR1232** | *Selaserica nitida* | Nuwara Eliya District | Horton Plains | L6 | BOLD:AEH6993 | MW698464 |

**Supplementary Figures**

Fig. S1. Maximum likelihood tree from PhyML analysis. Approximate likelihood ratio test (aLRT) values >0.5 are shown next to the respective branches.

Fig. S2. Split network of all examined specimens. Singletons are highlighted in blue squares, others in orange colors. Morphospecies nested within others are highlighted with red circles around them.
